# Physician Perceptions of Palliative Care for Children With Cancer in Latin America

**DOI:** 10.1001/jamanetworkopen.2022.1245

**Published:** 2022-03-08

**Authors:** Michael J. McNeil, Bella S. Ehrlich, Huiqi Wang, Yuvanesh Vedaraju, Marisol Bustamante, Veronica Dussel, Paola Friedrich, Ximena Garcia Quintero, Srinithya R. Gillipelli, Wendy Gomez Garcia, Dylan E. Graetz, Erica C. Kaye, Monika L. Metzger, Carla Vittoria Sabato Danon, Meenakshi Devidas, Justin N. Baker, Asya Agulnik

**Affiliations:** 1 Department of Global Pediatric Medicine, St Jude Children’s Research Hospital, Memphis, Tennessee; 2 Division of Quality of Life and Palliative Care Department of Oncology, St Jude Children’s Research Hospital, Memphis, Tennessee; 3 Brown University School of Medicine, Providence, Rhode Island; 4 Independent consultant, Guatemala City, Guatemala; 5 Center for Research and Implementation in Palliative Care, Institute for Clinical Effectiveness and Health Policy, Buenos Aires, Argentina; 6 Fundación Valle de Lilli, Cali, Colombia; 7 Baylor College of Medicine, Houston, Texas; 8 Oncology Unit, Dr Robert Reid Cabral Children’s Hospital, Santo Domingo, Dominican Republic; 9 Centro de Diagnóstico y Terapia Psicológica, Mexico City, Mexico

## Abstract

**Importance:**

The World Health Organization (WHO) designates early integration of palliative care as an ethical responsibility in the treatment of children with serious illness. Although structural barriers may influence provision of pediatric palliative care (PPC) for children with cancer in resource-limited settings, underlying physician perceptions may also impede early integration of PPC in cancer care.

**Objective:**

To investigate perceptions among physicians in Latin America about the integration of palliative care for children with cancer.

**Design, Setting, and Participants:**

This survey study used the Assessing Doctors’ Attitudes on Palliative Treatment (ADAPT) survey, which was developed for physicians who care for children with cancer and was initially distributed in Eurasia. The survey was modified for use in Latin America, including translation into Spanish and adaptation for cultural context. The survey was distributed between August 21, 2020, and January 31, 2021, to physicians treating children with cancer in 17 Latin American countries. Each country had a specific survey distribution method based on guidance of local experts.

**Main Outcomes and Measures:**

The ADAPT survey evaluated physicians’ understanding of palliative care principles, comfort in addressing patient and family suffering, and identification of barriers to PPC integration for children with cancer. Univariate and multivariable linear regression analyses were used to assess factors associated with physicians’ knowledge about and comfort with PPC practice and whether independent physician variables were associated with survey response alignment with WHO guidance on PPC. Open-ended questions were analyzed qualitatively to supplement the quantitative data.

**Results:**

A total of 874 physicians from 17 countries participated, with an overall response rate of 39.9% (874 of 2193) and a median country response rate of 51.4% (range, 23.7%-100%). Most respondents were aged 35 years or older (577 [66.0%]), and 594 (68.0%) identified as female. Most physicians (486 [55.6%]) had no formal PPC training, and 303 (34.7%) had no access to PPC experts for consultation. Physician perspectives on PPC were generally aligned with WHO guidance (mean [SD] alignment, 83.0% [14.1%]; range among respondents, 24.0%-100%). However, only 438 respondents (50.1%) felt comfortable addressing physical symptoms of patients receiving PPC, 295 (33.8%) felt comfortable addressing emotional symptoms, and 216 (24.7%) felt comfortable addressing grief and bereavement needs of the patient’s family. A total of 829 participants (94.8%) desired further education and training in PPC.

**Conclusion and Relevance:**

Although physicians’ perspectives aligned well with WHO guidance for PPC, this survey study identified opportunities for improving physician training in symptom management and emotional support for children with cancer and their families. These findings may inform the development of targeted interventions to improve the quality of PPC for children with cancer in Latin America.

## Key Points

**Question:**

What are the perceptions among physicians caring for children with cancer in Latin America on the integration of palliative care?

**Findings:**

In this survey study of 874 physicians from 17 countries in Latin America, physicians’ understanding of palliative care was generally aligned with World Health Organization guidance, but their comfort level in providing palliative care to patients and families was low.

**Meaning:**

The findings suggest that opportunities exist for improving physician training in symptom management and emotional support for children with cancer and their families.

## Teaser

This survey study of physicians in Latin America who care for children with cancer investigates their perceptions about and comfort with pediatric palliative care and how their perceptions align with World Health Organization guidance.

## Introduction

During the past 50 years, 5-year survival among children with cancer has greatly improved and now exceeds 80% in most high-income countries.^1^ However, more than 80% of children with cancer live in low- and middle-income countries, and most die of their disease.^2,3,4^ The World Health Organization (WHO) has stated that early integration of palliative care is an “ethical responsibility” in the treatment of children with life-threatening illness, including cancer, and should be a part of care regardless of available resources.^5,6^^(p2)^

The WHO defines palliative care as the prevention and relief of physical, psychosocial, and spiritual suffering among patients and their families facing life-threatening illness.^6^ Studies have demonstrated that early integration of palliative care in pediatrics helps with pain and symptom management, caregiver and patient suffering, and family communication.^7,8,9,10^ In low- and middle-income countries, access to pediatric palliative care (PPC) is limited; more than 65% of countries worldwide do not have PPC services.^11^ In addition, countries with the highest rates of child mortality and health-related suffering are those with the least access to PPC services.^12,13,14^

Although structural limitations, such as access to PPC specialists and financial barriers, affect the delivery of PPC, underlying perceptions among physicians also prevent early integration of PPC.^15,16,17^ Most research into physician perceptions of PPC has been conducted in high-income countries, but some recent studies have included low- and middle- income countries.^18,19,20^ However, the perceptions of PPC among physicians who care for children with cancer in Latin America are still unknown.

In collaboration with St Jude Global and the WHO Global Initiative for Childhood Cancer,^21,22^ the Assessing Doctors’ Attitudes on Palliative Treatment (ADAPT) survey was created to assess the perceptions of physicians caring for children with cancer regarding PPC integration. The survey was initially distributed to physicians in 11 countries in Eastern Europe and Central Asia.^18,19^ The objective of this study was to distribute the ADAPT survey to assess physicians’ perceptions of PPC and their comfort with delivering primary palliative care in 17 countries in Central and South America.

## Methods

This survey study was deemed exempt from the need for approval by the Office of Human Subjects Research Protections and Institutional Review Board at St Jude Children’s Research Hospital, Memphis, Tennessee, because the participants’ identity could not be readily ascertained. Additional approvals by participating institutions were obtained as needed. Voluntary completion of the survey was considered consent to participate in the study. This study followed the American Association for Public Opinion Research (AAPOR) reporting guideline.^23^

### Instrument Development and WHO Alignment

The ADAPT survey was initially developed to evaluate perspectives of physicians of all specialties who care for pediatric oncology patients in the Eurasian region.^18,19^ The survey items were developed based on WHO guidance and a review of published literature on physician perceptions of palliative care.^7,15,24,25,26^

The survey questions were translated into Spanish and evaluated by a team of 5 bilingual Latin American PPC and pediatric oncology experts (M.B., V.D., X.G.Q., W.G.G., and C.V.S.D.) who assessed the translated survey to ensure appropriate syntax, comprehension, and cultural relevance. The survey then underwent iterative rounds of review to improve content validity and cultural sensitivity. The survey was then translated back into English to ensure construct consistency.^27^ Next, the survey was piloted by 13 Spanish-speaking physicians representative of the target specialties for the survey. Adjustments were made based on the pilot participants’ feedback. The final survey contained 65 items, including 62 closed-ended items using a 5-point Likert scale ranging from 5 (“strongly agree”) to 1 (“strongly disagree”) or a multiple-choice format and 3 open-ended questions (eFigure in Supplement 1).

### Survey Distribution Strategy

The ADAPT survey was distributed anonymously in English and Spanish using the Qualtrics electronic software platform^28^ to physicians who treated children with cancer in 17 participating countries: Argentina, Bolivia, Chile, Colombia, Costa Rica, Dominican Republic, Ecuador, El Salvador, Guatemala, Haiti, Honduras, Mexico, Nicaragua, Panama, Paraguay, Peru, and Uruguay. Local collaborators designed the distribution strategy specific to each country’s physician workforce (eTable 1 in Supplement 1). In 2 countries (Argentina and Paraguay), there was a preexisting contact list of physicians who treated children with cancer; in the remaining countries, the survey was distributed by institutional leaders who identified colleagues at their institution. These institutional leaders were identified by regional and/or country leaders. Local collaborators recorded how many clinicians received the survey, and they sent a reminder email at least 1 week before the survey closed. The survey was distributed between August 21, 2020, and January 31, 2021, and was open at each site or country for 4 to 8 weeks.

### Statistical Analysis

Country-specific demographic data were reported using descriptive statistics. For calculating alignment with WHO guidance, 15 statements in the survey were categorized based on agreement or disagreement with the WHO guidance (eTable 2 in Supplement 1).^7,18^ A percentage-of-alignment (agreement) score was calculated for each respondent. The primary outcome was the mean percentage of alignment of the individual participants’ responses across the cohort. Univariate (simple linear regression analysis) and multivariable (multiple linear regression analysis) models were used to assess whether independent variables were significantly associated with WHO alignment scoring. All demographic factors and perspectives were included in the univariate analyses. Those for which the results were significant were then included in the multivariable analysis. Owing to small sample sizes for certain countries, all included countries were collapsed into World Bank income status groups.^29^ For secondary analyses, the 5-point Likert scale was collapsed into 3 categories (“often or always,” “sometimes,” and “never or rarely”) to compare associations with specific demographic variables by the Pearson χ^2^ or Fisher exact test. A 2-tailed *P* < .05 was considered statistically significant. SAS, version 9.4 (SAS Institute) was used for all summaries and analyses.^30^

In a qualitative analysis, written free-text responses to item 13, “What does palliative care mean to you?” were translated into English by bilingual members of the study team (M.J.M., S.R.G.). An initial codebook was deductively chosen based on previous ADAPT studies^18^ and supplemented inductively from iterative review of free-text responses (the final codebook is shown in eTable 3 in Supplement 1).^31^ The updated codebook was piloted by 2 coders (M.J.M., S.R.G.) to ensure consistent application of the codes. Each free-text response served as a unit of analysis. After double coding the first 20% of responses, the coders achieved a κ value of 0.81, demonstrating excellent interrater reliability. The remaining free-text codes were then divided between the 2 coders (M.J.M., S.R.G.) for coding, and thematic content analysis was performed to examine the role of palliative care in patient care as well as the timing of palliative care integration. All qualitative data analysis was performed using MAXQDA software.^32^

## Results

### Participant Demographics

The ADAPT survey was completed by 874 physicians from 17 countries in Latin America; the overall response rate was 39.9% (874 of 2193), and the median country response rate was 51.4% (range, 23.7%-100%) (eTable 1 in Supplement 1). Most respondents were aged 35 years or older (577 [66.0%]), identified as female (594 [68.0%]), and had more than 10 years of experience as a physician after graduating from medical school (479 [54.8%]). The most common specialty was general pediatrics (298 [34.1%]), with another 233 physicians (26.7%) being pediatric hematologist-oncologists (Table 1). Most participants practiced at a general hospital (390 [44.6%]). Most respondents (486 [55.6%]) had not received any previous palliative care training (types of PPC training are shown in eTable 4 in Supplement 1), and 303 (34.7%) did not have access to palliative care consultation. Of respondents who reported access to palliative care consultation, 555 (97.2%) reported access to a physician, with fewer reporting access to other multidisciplinary team members (psychologists, 147 [25.7%]; nurses, 112 [19.6%]; and social workers, 68 [11.9%]). Most respondents (769 [88.0%]) reported treating at least 1 patient who died in the previous year.

**Table 1.** Demographic Characteristics of Respondents to the Assessing Doctors’ Attitudes on Palliative Treatment Survey in Latin America

Characteristic	Respondents, No. (%) (N = 874)
Country	
Argentina	63 (7.2)
Bolivia	25 (2.9)
Chile	57 (6.5)
Colombia	98 (11.2)
Costa Rica	13 (1.5)
Dominican Republic	43 (4.9)
Ecuador	19 (2.2)
El Salvador	22 (2.5)
Guatemala	19 (2.2)
Haiti	11 (1.3)
Honduras	47 (5.4)
Mexico	192 (22.0)
Nicaragua	4 (0.5)
Panama	38 (4.3)
Paraguay	159 (18.2)
Peru	48 (5.5)
Uruguay	16 (1.8)
Age, y	
<35	297 (34.0)
≥35	577 (66.0)
Sex	
Female	594 (68.0)
Male	280 (32.0)
Primary medical specialty	
General pediatrics	298 (34.1)
Pediatric hematology-oncology	233 (26.7)
Pediatric palliative care	35 (4.0)
Other^a^	308 (35.2)
Primary institution	
General hospital	390 (44.6)
Children’s hospital	344 (39.4)
Cancer hospital	115 (13.2)
Other	25 (2.9)
Experience as a physician, y	
0-10	395 (45.2)
≥11	479 (54.8)
Trained in palliative care	
Yes	388 (44.4)
No	486 (55.6)
Access to palliative care consultation	
Yes	571 (65.3)
No	303 (34.7)
Patients who died during care in previous year, No.	
0	105 (12.0)
1-5	465 (53.2)
≥6	304 (34.8)

^a^ Other specialties included pediatric anesthesiology, pediatric surgery, pediatric intensive care, adult palliative care, general internal medicine and/or family medicine, adult hematology-oncology, adult anesthesiology, adult surgery, adult intensive care, pediatric infectious disease, pediatric subspecialty, surgical subspecialty, and other subspecialty.

### Components of Palliative Care

When asked the role of palliative care for children with cancer, most respondents picked all available options, including reducing suffering and improving quality of life, addressing communication needs, and assisting with end-of-life care (eTable 5 in Supplement 1). This finding was further expanded in the free-text response to the question “What does palliative care mean to you?” Respondents described palliative care as a specialty that addresses the patient’s quality of life by reducing suffering and addressing symptom burden: “health care aimed at promoting the decrease in suffering and improving the quality of life of the patient and his or her family.” Less commonly, some respondents described palliative care involvement in the psychological and spiritual elements of care: “comprehensive medical, emotional, and spiritual care of the patient, to avoid the suffering and pain that his/her illness/condition or treatment may cause.”

In addition, many respondents described the timing of palliative care involvement as the end of life, and the word *terminal* was frequently used to describe patients who would benefit from palliative care. Fewer respondents described the need for palliative care to be involved at the beginning of cancer care. Nevertheless, some respondents stated the importance of early integration: “multidisciplinary care provided to the patient for comprehensive care, which should be initiated once the patient is diagnosed with a life-threatening illness.” In addition, many of the respondents used the Spanish word *acompañar,* meaning to accompany, support, or journey with, to describe the role of palliative care as being with and accompanying patients and families during their cancer care and end of life: “actively accompany in the final stages of a terminal illness with the aim of maintaining a quality of life that respects a human being in a holistic and personalized way.”

### Alignment With WHO Guidance

For the 15 statements assessed for alignment with WHO guidance for PPC, the overall mean (SD) percentage of alignment was 83.0% (14.1%), with a range among respondents of 24.0% to 100%. In the univariate analyses, country income level, age, spirituality, primary medical specialty, years of experience as a physician, palliative care education, access to palliative care consultation, and greater experience with patient death (≥6 patient deaths in the previous year) were significantly associated with response alignment with WHO guidance. Multivariable analysis demonstrated that country income level, spirituality, and palliative care education were significantly associated with WHO alignment; however, the difference was small between groups (Table 2).

**Table 2.** Association Between Respondent Demographic Factors and Response Alignment With WHO Guidance for Pediatric Palliative Care

Factor	Mean WHO alignment, % (95% CI)	*P* value	
		Univariate model	Multivariable model^a^
Country’s income level			
Lower middle^b^	78.2 (75.6-80.9)	<.001	.01
Upper middle^c^	83.7 (82.7-84.8)		
High^d^	82.8 (79.6-86.1)		
Age, y			
<35 y	80.5 (78.9-82.1)	<.001	.24
≥35 y	84.2 (83.1-85.4)		
Sex			
Female	83.4 (82.3-84.5)	.20	NA
Male	82.1 (80.4-83.7)		
Religious			
Yes	82.8 (81.4-84.1)	.85	NA
Neutral	82.9 (81.0-84.8)		
No	83.4 (81.6-85.3)		
Spiritual			
Yes	83.9 (82.7-85.1)	.02	.03
Neutral	80.8 (78.9-82.7)		
No	82.4 (79.9-84.9)		
Primary medical specialty			
General pediatrics	81.1 (79.5-82.6)	<.001	.13
Pediatric hematology-oncology	85.0 (83.2-86.8)		
Pediatric palliative care	91.8 (87.2-96.4)		
Other^e^	82.3 (80.7-83.8)		
Primary institution			
General hospital	83.1 (81.7-84.5)	.08	NA
Children’s hospital	82.8 (81.4-84.3)		
Cancer hospital	81.3 (78.8-83.9)		
Other	89.3 (83.8-94.9)		
Experience as a physician, y			
0-10	81.1 (79.7-82.5)	<.001	.68
≥11	84.5 (83.2-85.7)		
Palliative care education			
Yes	86.0 (84.6-87.4)	<.001	<.001
No	80.6 (79.3-81.8)		
Access to palliative care consultation			
Yes	83.9 (82.8-85.1)	.005	.13
No	81.1 (79.5-82.7)		
Patients who died during care in previous year, No.			
0-5	82.1 (80.9-83.2)	.009	.06
≥6	84.7 (83.1-86.3)		

Abbreviations: NA, not applicable; WHO, World Health Organization.

^a^ Only independent variables that were statistically significant in the univariate analysis were included in the multivariable analysis.

^b^ Bolivia, El Salvador, Haiti, Honduras, and Nicaragua.

^c^ Argentina, Colombia, Costa Rica, Dominican Republic, Ecuador, Guatemala, Mexico, Panama, Paraguay, and Peru.

^d^ Chile and Uruguay.

^e^ Other specialties included pediatric anesthesiology, pediatric surgery, pediatric intensive care, adult palliative care, general internal medicine and/or family medicine, adult hematology-oncology, adult anesthesiology, adult surgery, adult intensive care, pediatric infectious disease, pediatric subspecialty, surgical subspecialty, and other subspecialty.

The most common misconceptions about palliative care were that (1) palliative care is synonymous with end-of-life care (43.4% of responses were not aligned with WHO guidance), (2) early consultation with palliative care causes increased parental burden and anxiety (36.8% were not aligned), and (3) it is difficult to know when a patient with cancer would most benefit from a consultation with the palliative care team (30.4% were not aligned) (eTable 6 in Supplement 1).

### Comfort Delivering Palliative Care and Feeling Burdened

Although response alignment with WHO guidance was high, physician comfort in delivering components of palliative care was lower. Overall, only 438 respondents (50.1%) felt comfortable addressing the physical needs of patients, and even fewer felt comfortable addressing patients’ emotional needs (295 [33.8%]) and spiritual needs (255 [29.2%]) or the grief and bereavement needs of family members (216 [24.7%]) (Figure). Previous palliative care training was associated with physician comfort in addressing patient needs (Table 3). Whereas 251 of the 388 physicians with palliative care training (64.7%) felt comfortable addressing the physical needs of patients, only 187 of the 486 physicians without palliative training (38.5%) felt comfortable (*P* < .001). Similarly, more physicians with previous palliative care training vs those without training reported feeling comfortable addressing emotional needs (189 [48.7%] vs 106 [21.8%]; *P* < .001), spiritual needs (156 [40.2%] vs 99 [20.4%]; *P* < .001), and grief and bereavement needs (137 [35.3%] vs 79 [16.3%]; *P* < .001). In addition, 95 physicians with previous palliative care training (24.5%) felt overburdened by patients’ suffering compared with 183 physicians without training in palliative care (37.7%) (*P* < .001).

**Figure.**
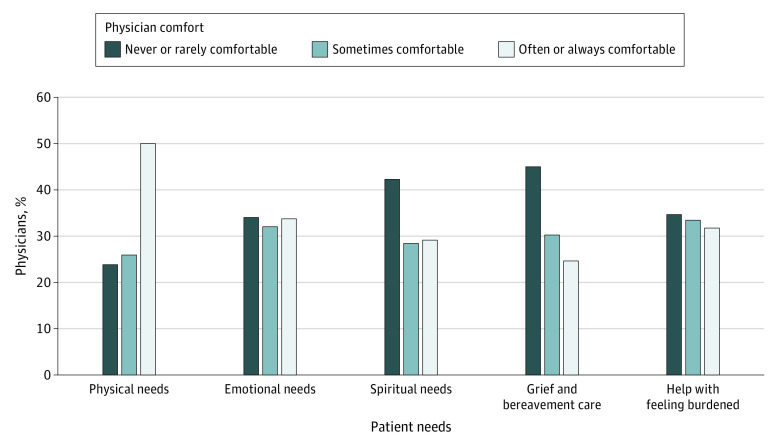
Physician Attitudes Toward Addressing Needs of Patients Receiving Pediatric Palliative Care and Their Families Participants responded to survey items using a 5-point Likert scale collapsed into 3 categories.

**Table 3.** Physician Responses to Survey Items by Whether They Received Palliative Care Training

Survey item	Previous palliative care training, No. (%)		*P* value^a^
	Yes (N = 388)	No (N = 486)	
I feel confident assessing and treating the physical needs of pediatric patients with serious incurable illness			
Never or rarely	59 (15.2)	150 (30.9)	<.001
Sometimes	78 (20.1)	149 (30.7)	
Often or always	251 (64.7)	187 (38.5)	
I feel confident assessing and treating the emotional needs of pediatric patients with serious incurable illness and their families			
Never or rarely	84 (21.6)	214 (44.0)	<.001
Sometimes	115 (29.6)	166 (34.2)	
Often or always	189 (48.7)	106 (21.8)	
I feel confident taking care of the spiritual needs of pediatric patients with serious incurable illness and their families			
Never or rarely	116 (29.9)	254 (52.3)	<.001
Sometimes	116 (29.9)	133 (27.4)	
Often or always	156 (40.2)	99 (20.4)	
I feel confident providing grief and bereavement care to the families of children who die			
Never or rarely	122 (31.4)	271 (55.8)	<.001
Sometimes	129 (33.2)	136 (28.0)	
Often or always	137 (35.3)	79 (16.3)	
I have felt burdened by my inability to control the suffering of children at the end of life			
Never or rarely	149 (38.4)	154 (31.7)	<.001
Sometimes	144 (37.1)	149 (30.7)	
Often or always	95 (24.5)	183 (37.7)	

^a^ χ^2^ test.

### Palliative Care Practices and Attitudes

Many respondents believed that quality of life was often overlooked in cancer treatment (644 [73.9%]), with 366 (41.9%) stating that palliative care involvement occurred too late and 329 (37.7%) reporting that physicians continued to recommend cancer-directed treatment for children with incurable cancer even when that treatment was ineffective or unlikely to prolong a child’s life. Only 501 respondents (57.3%) felt that palliative care consultation was available when it was needed in their setting. Most respondents (842 [96.3%]) felt that a greater emphasis on palliative care education for health care professionals is an important step in improving access to palliative care, with 829 (94.8%) stating they wished to have more education on providing palliative care for patients (eTable 7 in Supplement 1).

## Discussion

The early integration of palliative care for patients with life-threatening illnesses, including cancer, is an ethical responsibility for clinicians regardless of resources.^6,7^ Although structural and financial barriers exist, a lack of knowledge of palliative care among clinicians may impede early integration of PPC for children with cancer.^7^ The findings of this survey study demonstrated that although physicians in Latin America had accurate knowledge of PPC that was aligned with WHO guidance in general, there was a need for PPC education, as evidenced by the percentage of physicians who reported low confidence in treating the physical, emotional, spiritual, and bereavement needs of patients and their families.

It is expected that physician knowledge and experience differ across regions, which is supported by differences seen between this study and the initial ADAPT survey findings.^18,19^ Although the alignment of participants’ responses with WHO guidance on PPC was similar between Latin America (83.0%) and Eurasia^18^ (70%), the reported comfort level in addressing patients’ palliative care needs was lower in Latin America. Of interest, whereas physicians in Eurasia^18^ felt more comfortable addressing patient needs, more (62%) felt overburdened by their inability to address patient suffering than in the Latin American cohort in the present study (37.7%). These differences may be associated with access to PPC or previous palliative care training (both were more common in Latin America than in Eurasia), but it is also possible that comfort level does not equal competency. These findings support the importance of conducting this research in different settings and countries so as not to assume all low- and middle-income countries experience identical challenges to PPC integration. Further research is needed.

A prior study^20^ evaluating the knowledge and comfort in providing PPC among Mexican pediatricians demonstrated that pediatricians with previous palliative care training and exposure and those who cared for children with cancer had greater knowledge and comfort in addressing the PPC needs of patients. Our findings corroborate those results across the region, with professional specialty and palliative care training being associated with PPC knowledge and comfort. These findings emphasize the importance of region-specific palliative care education for physicians caring for children with cancer to improve the quality of care provided to all children in the region.

Although high alignment of survey responses with WHO guidance on PPC suggests that physicians had a good understanding of basic PPC principles, our study found that physicians were not comfortable addressing patients’ physical, emotional, and spiritual needs or the grief and bereavement of families. In addition, there were some misconceptions identified in the survey, such as that palliative care is synonymous with end-of-life care (43.4% of respondents), which was reported by a similar percentage of a cohort of pediatricians in the US (44%).^15^ Initiatives and interventions in Latin America should decouple the conceptualization of palliative care as the same as end-of-life care, promote its earlier integration into childhood cancer care, and teach tools to address patient and family palliative care needs. The ADAPT findings informed development of educational initiatives in Eurasia, and these findings can similarly direct educational initiatives in Latin America. The perception that palliative care is accompanying the patients through their journey can be leveraged to better align timing of palliative care involvement earlier in the patient’s treatment course.

Supporting the need for education, our study found an association between PPC education and physicians feeling overburdened. Feeling overburdened by one’s work is an important component of burnout and compassion fatigue.^33,34^ In this study’s specific context, feeling burdened by the lack of skills to care for patients at the end of life may have been managed by having tools to address patient suffering through palliative care education. This may have important implications for addressing burnout and compassion fatigue for physicians caring for children with cancer in Latin America.^35,36^

Beyond informing educational efforts, results from this study will be used to advise local leaders in Latin America on educational priorities to improve PPC for children with cancer. In Eurasia, a 2-page report for each country summarizing the results of the ADAPT study and identifying tangible opportunities for intervention was developed to inform local institutional and ministry of health leaders.^37^ Similarly, we will synthesize country-level data from our study to provide tools to local leaders to advocate for improvements in PPC based on their country’s specific needs.

### Limitations

This study has several limitations. First, owing to language differences, the survey was distributed in primarily Spanish-speaking countries. However, the survey was also translated into Portuguese with plans to distribute it in Brazil. Also, owing to the differences in health care infrastructure and physician workforce across countries in the region, different survey distribution strategies were used to tailor distribution to individual countries’ resources and needs. On the basis of our experience with ADAPT in Eurasia, we attempted to distribute the survey by institution instead of via large listservs, but our ability to do so was contingent on local resources and capacity. This strategy resulted in disproportionate responses as they pertained to country population. Although this method of distribution resulted in some institutions not participating owing to lack of capacity and the potential exclusion of some eligible participants, to our knowledge, this work was the largest study to date on this topic. The large sample size of respondents makes us confident that our findings are representative of physicians caring for children with cancer in participating countries. Moreover, although there were some statistically significant differences in the multivariable analysis, the true percentage differences were small and may not represent a clinically significant difference in physician knowledge. Therefore, a focus on the independent variables with larger differences in alignment, such as palliative care education, is warranted. In addition, the survey was distributed electronically, which may have limited participation of physicians in more rural locations, potentially biasing our results toward more well-resourced institutions in urban areas, where palliative care knowledge and access is likely to be higher. However, childhood cancer care in Latin America is typically centralized to these institutions, and we believe our findings are representative of perspectives and knowledge of physicians responsible for most pediatric oncology care in the region.

## Conclusions

The findings of this survey study provide important insights on the perceptions of PPC among physicians caring for children with cancer in Latin America. Although physician knowledge was aligned with WHO guidance on PPC in general, further education appears to be needed to promote early integration of PPC for children with cancer in the region. Also, regionally adapted educational interventions appear to be needed to improve physicians’ abilities to address patients’ physical, emotional, and spiritual needs along with grief and bereavement care for families. These initiatives should be offered to all physicians who care for children with cancer, including in specialties other than pediatric hematology-oncology. These regional interventions may provide physicians with resources to reduce their sense of burden and risk of burnout and improve their ability to reduce the suffering of patients and families.

## Notes

Supplement 1. **eFigure.** ADAPT Latin America Survey **eTable 1.** Country-specific Distribution Method **eTable 2.** WHO ADAPT Questions **eTable 3.** ADAPT Latin America Qualitative Codebook **eTable 4.** Types of Pediatric Palliative Care Training **eTable 5.** The Components of Palliative Care **eTable 6.** Percent Aligned With WHO Guidance: Most to Least Common **eTable 7.** Provider Attitudes Toward Palliative Care (N = 874)

Supplement 2.
Assessing Doctors’ Attitudes on Palliative Treatment (ADAPT) Latin America Study Group
