# Supplementary material for: Physician Perceptions of Palliative Care for Children With Cancer in Latin America
Source: JAMA Netw Open. 2022 Mar 8;5(3):e221245. doi: 10.1001/jamanetworkopen.2022.1245 (PMC8905380; doi:10.1001/jamanetworkopen.2022.1245)
Supplement: Supplement 1. — eFigure. ADAPT Latin America Survey eTable 1. Country-specific Distribution Method eTable 2. WHO ADAPT Questions eTable 3. ADAPT Latin America Qualitative Codebook eTable 4. Types of Pediatric Palliative Care Training eTable 5. The Components of Palliative Care eTable 6. Percent Aligned With WHO Guidance: Most to Least Common eTable 7. Provider Attitudes Toward Palliative Care (N = 874) [file jamanetwopen-e221245-s001.pdf]

Supplementary Online Content

McNeil MJ, Ehrlich BS, Wang H, et al; Assessing Doctors' Attitudes on Palliative Treatment (ADAPT) Latin America Study Group. Physician perceptions of palliative care for children with cancer in Latin America. *JAMA Netw Open*. 2022;5(3):e221245. doi:10.1001/jamanetworkopen.2022.1245

eFigure. ADAPT Latin America Survey
eTable 1. Country-specific Distribution Method
eTable 2. WHO ADAPT Questions
eTable 3. ADAPT Latin America Qualitative Codebook
eTable 4. Types of Pediatric Palliative Care Training
eTable 5. The Components of Palliative Care
eTable 6. Percent Aligned With WHO Guidance: Most to Least Common
eTable 7. Provider Attitudes Toward Palliative Care (N = 874)

This supplementary material has been provided by the authors to give readers additional information about their work.

© 2022 McNeil MJ et al. *JAMA Network Open*.

Supplemental Tables and Figures:

Physician Perceptions of Pediatric Palliative Care for Children with Cancer in Latin America

Michael J. McNeil[1,2], Bella Ehrlich[1,3], Huiqi Wang[1], Marisol Bustamante[4], Veronica Dussel[5,] Paola Friedrich[1], Ximena Garcia Quintero[1,6], Srinithya R Gillipelli[1], Wendy Gomez Garcia[7], Dylan Graetz[1], Erica C. Kaye[2], Monika Metzger[1], Carla Vittoria Sabato Danon[8], Yuvanesh Vedaraju[1], Meenakshi Devidas[1], Justin N. Baker[2], Asya Agulnik[1] on behalf of the ADAPT LA study group

Table of Contents

Item	Page
**eFigure 1:** ADAPT LA Survey	2
**eTable 1:** Country-Specific Distribution Method	9
**eTable 2:** WHO ADAPT Questions	10
**eTable 3:** ADAPT LA Qualitative Codebook	11
**eTable 4:** Types of Pediatric Palliative Care Training	14
**eTable 5:** The Components of Palliative Care	14
**eTable 6:** Percent Correct with WHO Guidance: Most to Least Common	16
**eTable 7:** Provider attitudes toward palliative care	17

© 2022 McNeil MJ et al. *JAMA Network Open*.

eFigure 1: ADAPT Latin America100- Survey

Demographics

1.“Do you treat children with cancer as part of your medical practice?” (Yes/no)

2. What is the country in which you currently practice medicine?

- Argentina
- Bolivia
- Brazil
- Chile
- Colombia
- Costa Rica
- Dominical Republic
- Ecuador
- El Salvador
- Guatemala
- Haiti
- Honduras
- Mexico
- Nicaragua
- Panama
- Paraguay
- Peru
- Uruguay

3. Your age:

- <35 years
- 35 to 50 years
- 51 to 65 years
- >65 years

4. Your gender:

- Female
- Male
- Other
- Prefer not to disclose

5. Would you consider yourself a religious person? (religious: the search for significance that occurs within the context of established faith traditions (Judaism, Christianity, Muslim, Hindu, Buddhism, etc.)

- Strongly Disagree
- Disagree
- Uncertain
- Agree
- Strongly Agree

© 2022 McNeil MJ et al. *JAMA Network Open*.

6. Would you consider yourself a spiritual person? (spiritual: individual search for meaning and purpose)

- Strongly Disagree
- Disagree
- Uncertain
- Agree
- Strongly Agree

7. Please indicate your primary medical specialty:

- General pediatrician
- Pediatric hematology and/or oncology
- Pediatric anesthesiology
- Pediatric surgery
- Pediatric intensive care
- Pediatric Palliative care
- Adult Palliative Care
- General Internal Medicine/Family Medicine
- Adult hematology and/or oncology
- Adult anesthesiology
- Adult surgery
- Adult intensive care
- Other (describe)

8. How many years of experience in practice do you have since graduating medical school?

- 0-5 years
- 6-10 years
- 11-15 years
- 16-20 years
- 21+ years

9. Concerning the primary institution in which you work, indicate if it is a:

- General Hospital
- Children's Hospital
- Cancer Hospital
- Other (describe)

10. Have you received any formal training in palliative care?

- Yes
- No

If Yes (check all that apply):

- Continuing Medical Education (or postgraduate course without official certification)
- Certificate course
- Medical School or Post-graduate Rotation

© 2022 McNeil MJ et al. *JAMA Network Open*.

Undergraduate/ Medical School Course
Master's in Palliative Care
Residency or Fellowship in palliative care
Other: (Please describe)

11. Do you have access to a pediatric palliative care expert for consultation in your practice? (Yes/No)
If yes, please check the type(s) of palliative care experts available:
Physician, nurse, social worker, psychologist, other (please describe)

12. How many pediatric patients in your care (less than 18 years old) died in the last 12 months?
a. 0 patients
b. 1-5 patients
c. 6-10 patients
d. 11-20 patients
e. 21+ patients

13. What does palliative care mean to you?

Perspectives
Please rate the extent to which you agree with the following statements about ***pediatric oncology patients*** *receiving* ***palliative care****. Please check one box per line:*

	Strongly Disagree	***Somewhat Disagree***	***Neutral***	***Somewhat Agree***	***Strongly Agree***

14. It is difficult to know when a patient with cancer would most benefit from meeting the palliative care team.

15. Quality of life is often overlooked in the face of cancer-directed treatment.

16. Children with advanced and incurable cancer often suffer at the end-of-life.

17. Early consultation with palliative care causes increased parental burden and anxiety.

18. Palliative care is perceived by parents as meaning the end of life is near and that the oncologist will 'give up' on their child.

19. Palliative care can be integrated with disease-directed therapy.

20. Involving palliative care suggests the oncologist has failed in the mission to cure the patient.

21. There are situations where it is in a dying child's best interest to remove mechanical ventilation if in alignment with the family's wishes.

© 2022 McNeil MJ et al. *JAMA Network Open*.

22. Palliative care for children with cancer can be delivered by health care workers of all disciplines, not only by palliative care specialists.

23. Palliative care is synonymous with “end-of-life” care.

24. Involvement of palliative care during cancer therapy gives greater attention to quality of life and symptom management (e.g. pain, constipation, dyspnea, fatigue).

25. Involvement of palliative care undermines the role of the pediatric oncologist as the physician in charge of patient care.

26. Children with cancer who receive palliative care die earlier than those who do not.

27. Early integration of palliative care for all children diagnosed with cancer would decrease patient suffering.

28. Early integration of pediatric palliative care with cancer care would improve interdisciplinary communication.

29. Palliative care is incompatible with curative care.

30. Involving the palliative care team early has negative effects on the relationship between the oncologist and the patient and family.

31. Palliative care is appropriate at any stage of treatment in a child with high-risk cancer.

32. Administering opioids to patients in pain hastens death due to respiratory depression.

33. In my setting, physicians typically continue to recommend cancer-directed treatment for children with incurable oncological disease even when that treatment is ineffective or unlikely to prolong a child’s life.

34. In my setting, doctors generally feel confident taking care of the physical needs of pediatric patients with serious incurable illness.

35. In my setting, doctors generally feel confident taking care of the emotional needs of pediatric patients with serious incurable illness.

36. In my setting, doctors generally feel confident taking care of the spiritual needs of pediatric patients and their families with serious incurable illness.

37. In my setting, doctors generally feel confident providing grief and bereavement care to the families of children who die.

38. Greater emphasis on palliative care education for doctors and health care professionals is an important step in improving access to palliative care.

© 2022 McNeil MJ et al. *JAMA Network Open*.

39. I wish to have more education on how to provide palliative care to my patients.

Individual Experience:
*Please rate the **frequency** of the following statements about **your individual experiences** with **pediatric oncology patients**. Please check one box per line:*

	Never	*Rarely*	*Sometimes*	*Often*	*Always*

40. In my setting of practice, palliative care consultation is available when I feel it is needed for a child with cancer.

41. In my setting, I have felt that involvement of palliative care has occurred too late in the treatment of a child with cancer.

42. In my setting, I have acted against my conscience by providing **aggressive treatment** to a pediatric oncology patient with advancing disease.

43. I feel confident assessing and treating the **physical needs** of pediatric patients with serious incurable illness.

44. I feel confident assessing and treating the **emotional needs** of pediatric patients with serious incurable illness and their families.

45. I feel confident taking care of the **spiritual needs** of pediatric patients and their families with serious incurable illness.

46. I feel confident providing grief and bereavement care to the families of children who die.

47. I have felt burdened by my inability to control the suffering of children at the end-of-life.

Multiple choice:
*Please choose **all** that apply for every option.*

48. The role of palliative care in the care of children with cancer is (please choose all that apply):
- To aid in reducing pain and suffering related to disease and/or treatment
- To provide psychological support to the patient and their family
- To provide spiritual support to the patient and their family
- To aid in family decision-making around treatment options
- To help clarify the goals of care of the patient and family
- To help communicate bad news to patients and families
- To aid in communication between the patient, family, and medical teams
- To assist with transitions from the hospital to hospice or home at end-of-life
- Other: Please describe

© 2022 McNeil MJ et al. *JAMA Network Open*.

49. When does **initial** palliative care consultation for a child with cancer **typically** occur in your setting (please choose all that apply):

- At the time of cancer diagnosis for all patients
- At the time of cancer diagnosis for patients at high-risk of relapse or progression
- At the time of disease relapse or progression
- At the time of complex or high symptom burden (pain, suffering)
- When there are no longer curative therapeutic options available
- At the end of life
- Palliative care is typically not consulted for children with cancer (because it is not necessary or not available)

50. Assuming unlimited resources, when do you think is the **ideal** timing of **initial** palliative care consultation for a child with cancer (please choose all that apply):

- At the time of cancer diagnosis for all patients
- At the time of cancer diagnosis for patients at high-risk of relapse or progression
- At the time of disease relapse or progression
- At the time of complex or high symptom burden (pain, suffering)
- When there are no longer curative therapeutic options available
- At the end of life
- Palliative care consultation is never necessary in pediatric cancer care

51. If there is a difference between when initial palliative care consultation **typically occurs** in your setting and what you think is **ideal**, why do you think this difference exists?

Barriers*:*
Please rate the extent to which you feel the following statements represent barriers to ***early integration of palliative care for pediatric oncology patients****. Please check one box per line:*

	Extremely Unimportant Barrier	***Somewhat Unimportant Barrier***	***Neither Important nor Unimportant Barrier***	***Somewhat Important Barrier***	***Extremely Important Barrier***

52. Limited physician knowledge on the role of palliative care

53. Physician discomfort in raising the topic of palliative care with families

54. Physician desire to maintain hope

55. Uncertainty about patient prognosis

56. Family resistance to involvement of palliative care

57. Time constraints of pediatric oncologists during consultation

© 2022 McNeil MJ et al. *JAMA Network Open*.

58. Lack of home-based services

59. Limited access to opioids

60. Limited access to palliative care specialists or services

61. Cost of palliative care consultation and treatment

62. Cultural differences between patients/families and physicians

63. Differences in languages between patients/families and physicians

64. Are there barriers to early integration of palliative care for pediatric oncology patients not listed above? If yes, please identify the barriers and rate their importance below. If no additional barriers exist, go to the next question.

65. Do you have any additional comments or concerns regarding palliative care for children and adolescents with cancer?

eTable 1: Country-Specific Distribution Method

© 2022 McNeil MJ et al. *JAMA Network Open*.

Country	Distribution Method	Number of surveys distributed, No.	Number of surveys completed, No.	Response Rate, %
**Argentina**	Distribution by formalized country contact list	266	63	24%
**Bolivia**	Distribution by institution contact list	52	25	48%
**Chile**	Distribution by institution contact list	184	57	31%
**Colombia**	Distribution by institution contact list	321	98	31%
**Costa Rica**	Distribution by institution contact list	42	13	31%
**Dominican Republic**	Distribution by institution contact list	55	43	78%
**Ecuador**	Distribution by institution contact list	37	19	51%
**El Salvador**	Distribution by institution contact list	22	22	100%
**Guatemala**	Distribution by institution contact list	35	19	54%
**Haiti**	Distribution by formalized country contact list	21	11	52%
**Honduras**	Distribution by institution contact list	52	47	90%
**Mexico**	Distribution by institution contact list	585	192	33%
**Nicaragua**	Distribution by institution contact list	6	4	67%
**Panama**	Distribution by institution contact list	75	38	51%
**Paraguay**	Distribution by formalized country contact list	339	159	47%
**Peru**	Distribution by institution contact list	83	48	58%
**Uruguay**	Distribution by institution contact list	18	16	89%
**Total**		2,193	874	40%
**Median Country Response Rate**				**52%**

© 2022 McNeil MJ et al. *JAMA Network Open*.

eTable 2: WHO ADAPT Questions

Perspective Statement	WHO Alignment
16. Children with advanced and incurable cancer often suffer at the end-of-life.	Agree
19. Palliative care can be integrated with disease-directed therapy.	Agree
22. Palliative care for children with cancer can be delivered by health care workers of all disciplines, not only by palliative care specialists.	Agree
24. Involvement of palliative care during cancer therapy gives greater attention to quality of life and symptom management (e.g. pain, constipation, dyspnea, fatigue).	Agree
27. Early integration of palliative care for all children diagnosed with cancer would decrease patient suffering.	Agree
28. Early integration of pediatric palliative care with cancer care would improve interdisciplinary communication.	Agree
31. Palliative care is appropriate at any stage of treatment in a child with high-risk cancer.	Agree
14. It is difficult to know when a patient with cancer would most benefit from meeting the palliative care team.	Disagree
17. Early consultation with palliative care causes increased parental burden and anxiety.	Disagree
20. Involving palliative care suggests the oncologist has failed in the mission to cure the patient.	Disagree
23. Palliative care is synonymous with “end-of-life” care.	Disagree
25. Involvement of palliative care undermines the role of the pediatric oncologist as the physician in charge of patient care.	Disagree
26. Children with cancer who receive palliative care die earlier than those who do not.	Disagree
29. Palliative care is incompatible with curative care.	Disagree
30. Involving the palliative care team early has negative effects on the relationship between the oncologist and the patient and family.	Disagree

© 2022 McNeil MJ et al. *JAMA Network Open*.

eTable 3: ADAPT Latin America Qualitative Codebook

Category	Code	Definition
Components/Role of Palliative Care		
	Accompany	Refers to the role of the palliative care provider/team to accompany the patient and their family in experiencing a serious or life-threatening illness.
	Psychological Support	Refers to palliative care as any general psychological support, assistance, and care provided by the physician or palliative care team to the patient and/or family. This is separate from the code "quality of life for patient" that describes a patient's emotional state (e.g. key words include "emotional", "moral"). This includes grief and bereavement, and any psychological support provided to families around the dying process, either at the end-of-life or after the passing of the patient.
	Patient Quality of Life	Reference to palliative care as the quality of life of the patient, including any mention of "suffering", as well as "comfort". This includes mention of "maximum" benefit and "individualized care" to the patient. This about this code when using phrases such as "relieving", "easing", "facilitating", "improving", and "ameliorating". Do NOT code vague terms such as "caring", "helping" or "supporting" if there is no context.
	Medical Care	Refer to palliative care as medical treatment or medical care, not including symptom management. This includes any mention of "procedures", "interventions", "treatment", "medical care", as well body processes ("functions", "vitals"). Does NOT include management of symptoms or caring for the "physical condition" (implying symptoms), such as pain (code under "Symptom Management").
	Symptom Management	Any reference to palliative care as symptom therapy or pain management, rehabilitation, and improvement of "pain" or the "physical condition". Includes any mention of "maintenance". Does NOT include mentions of "suffering" only (code "Patient quality of Life"). Further, does NOT include any medical care or treatment not directed at symptoms (code as "Medical care").
	Life Extension	Any references to palliative care as involving the lengthening of life (key words include "prolongation", "extension"). This does NOT include references of solely lengthening moments of high quality of life/symptom management within the same course of a patient's life.
	End-of-life Care	Any references to palliative care as end-of-life care, death, or passing away. This includes mentions of easing the process of death, as well normalization and acceptance of death for either patient or family (e.g. any mention of "death" and "passing away") . DO include "until end-of-life", implying end-of-life is a component of palliative care.
	Religious/Spiritual Care	Refers to palliative care as the provision of spiritual or religious support to the patient and/or family.

© 2022 McNeil MJ et al. *JAMA Network Open*.

	Teamwork	Reference to palliative care as interdisciplinary communication within one institution, or collaboration between institutions. This includes specific references to involvement of nurses, psychologists, or different specialists, as well as transition from the hospital to hospice or home at the end-of-life.
	Parent Quality of Life	References to palliative care as any communication delivered by the doctor to the patient and/or family, whether it be the communication of bad news, aid in family decision-making around treatment options, or helping clarify the goals of care of the patient and family. This includes mention of the LACK of communication with the family on these topics. This does NOT include communication/refusal of care by the family in response to provider communication (code “society attitudes”).
Timing of Palliative Care		
	At diagnosis	This references the timing of palliative care involvement at the time of diagnosis. Refers to a specific time point when palliative care is integrated. This does NOT imply exclusivity with curative treatment.
	Disease Progression	Includes references to any mention of specific time points of palliative care integration or involvement that are not at diagnosis, at the end-of-life, or after treatment options are exhausted. This includes involvement given poor prognosis for high-risk patients, disease relapse or progression, and high symptom burden (pain, suffering). This does not imply exclusivity with curative treatment. Does NOT include referencing palliative care using an individualized approach or an on-need basis, unless there are further specifications about timing in the course of treatment/life.
	Integration with Treatment	Any references to palliative care to be integrated concurrently with treatment, with NO specific mention a time point, such as at diagnosis (code as "At diagnosis") or disease relapse/high symptom burden (code as "Disease Progression"). Key phrases such as "early integration" included here. Note, this is mutually exclusive with other timing codes.
	No other options available	This references the timing of palliative care involvement as conditional upon patient unresponsiveness to treatment and/or other treatment options being unavailable and "exhausted" (e.g. "treatment is failing", "no options available") . Refers to a specific time point when palliative care is integrated or involved in a patient's disease course. Often there is an implication of mutual exclusivity between curative therapy and symptomatic treatment/palliative care. DO include any mention of "incurable" disease/patients under this code.
	End-of-life/Terminal Stages	Includes any references to the specific time points of palliative care integration or involvement at the end-of-life of a patient. This also includes grief and bereavement support after death. Consider this code when seeing terms such as a "last resort", "last days of life", or "final landing". Any reference to care delivered to terminal patients, with no indication of previous exposure, is coded (e.g. "to improve quality of life of end-stage patients"). However, do NOT code words "until end-of-life" as it leaves open-ended when palliative care was initiated (code "end-of-life care" under component of

© 2022 McNeil MJ et al. *JAMA Network Open*.

		palliative care). This will often be double-coded with "end-of-life care".
Positive Attitudes		
	Celebration of Life	Refers to any comment on palliative care or hospice as a celebration of life. Examples include: "house of life", "better" or "decorating" life.
	Compassion and Love	Refers to the provision of compassion and love to the patient and/or family, including key words such as "care", "love", or "empathy".
	Necessary Service	Refers to palliative care as a mandatory service for any indicated population. Key words include "mandatory", "crucial", "integral", "necessary", "need", “comprehensive care” or "important". This further includes palliative care referred to as a right of the patients, and any references to palliative care being a free or government-funded service should be included in this code.
	Life/Death with Dignity	References to palliative care or hospice using any mention of the word "dignity" or "worth" over the course of the patient's life and/or death.

© 2022 McNeil MJ et al. *JAMA Network Open*.

eTable 4: Types of Pediatric Palliative Care Training

**Types of Palliative Care Training**	N=388 (%)
Continuing medical education	218 (56.2%)
Certificate course	104 (26.8%)
Medical school or post-graduate rotation	83 (21.4%)
Undergraduate/Medical school course	70 (18.0%)
Master’s in palliative care	26 (6.7%)
Residency or Fellowship in palliative care	30 (7.7%)
*Other	2 (0.5%)

*Other: Pain fellowship
Respondents could select more than one option with regards to training

eTable 5: The Components of Palliative Care

**The role of palliative care in the care of children with cancer is (choose all that apply):**	N= 874 (%)
To provide psychological support to the patient and their family	785 (89.8%)
To aid in reducing pain and suffering related to disease and/or treatment	784 (89.7%)
To aid in communication between the patient	779 (89.1%)
To help communicate bad news to patients and families	778 (89.0%)
To help clarify the goals of care of the patient and family	776 (88.8%)
To assist with transitions from the hospital to hospice or home at end-of-life	770 (88.1%)
To provide spiritual support to the patient and their family	768 (87.9%)
To aid in family decision-making around treatment options	744 (85.1%)

© 2022 McNeil MJ et al. *JAMA Network Open*.

eTable 6: Percent Aligned with WHO Guidance: Most to Least Common

Question	% Aligned
Involvement of palliative care during cancer therapy gives greater attention to quality of life and symptom management (e.g., pain, constipation, dyspnea, fatigue)	96.1%
Early integration of palliative care for all children diagnosed with cancer would decrease patient suffering	96.1%
Early integration of pediatric palliative care with cancer care would improve interdisciplinary communication	94.7%
Involving palliative care suggests the oncologist has failed in the mission to cure the patient	94.2%
Involvement of palliative care undermines the role of the pediatric oncologist as the physician in charge of patient care	92.8%
Palliative care is appropriate at any stage of treatment in a child with high-risk cancer	88.9%
Involving the palliative care team early has negative effects on the relationship between the oncologist and the patient and family	87.2%
Palliative care is incompatible with curative care	85.1%
Palliative care can be integrated with disease-directed therapy	84.8%
Children with cancer who receive palliative care die earlier than those who do not	83.9%
Palliative care for children with cancer can be delivered by health care workers of all disciplines, not only by palliative care specialists	76.2%
Children with advanced and incurable cancer often suffer at the end-of-life.	75.2%
It is difficult to know when a patient with cancer would most benefit from a consult with the palliative care team.	69.6%
Early consultation with palliative care causes increased parental burden and anxiety	63.2%
Palliative care is synonymous with "end-of-life" care	56.6%

© 2022 McNeil MJ et al. *JAMA Network Open*.

eTable 7: Provider attitudes toward palliative care (N=874)

Question	No (%)
**Quality of life is often overlooked in the face of cancer-directed treatment**	
Disagree	162 (18.5%)
Neutral	66 (7.6%)
Agree	644 (73.9%)
Missing	2
**In my setting, I have felt that involvement of palliative care has occurred too late in the treatment of a child with cancer.**	
Disagree	251 (28.7%)
Neutral	257 (29.4%)
Agree	366 (41.9%)
**In my setting, physicians typically continue to recommend cancer-directed treatment for children with incurable oncological disease even when that treatment is ineffective or unlikely to prolong a child's life**	
Disagree	317 (36.4%)
Neutral	226 (25.9%)
Agree	329 (37.7%)
Missing	2
**In my setting of practice, palliative care consultation is available when I feel it is needed for a child with cancer.**	
Disagree	235 (26.9%)
Neutral	138 (15.8%)
Agree	501 (57.3%)
**Greater emphasis on palliative care education for doctors and health care professionals is an important step in improving access to palliative care.**	
Disagree	17 (2.0%)
Neutral	9 (1.0%)
Agree	842 (97.0%)
Missing	6
**I wish to have more education on how to provide palliative care to my patients.**	
Disagree	17 (2.0%)
Neutral	21 (2.4%)
Agree	829 (95.6%)
Missing	7

© 2022 McNeil MJ et al. *JAMA Network Open*.
